# Multiplexing Methods for Simultaneous Large‐Scale Transcriptomic Profiling of Samples at Single‐Cell Resolution

**DOI:** 10.1002/advs.202101229

**Published:** 2021-07-08

**Authors:** Junyun Cheng, Jie Liao, Xin Shao, Xiaoyan Lu, Xiaohui Fan

**Affiliations:** ^1^ Pharmaceutical Informatics Institute College of Pharmaceutical Sciences Zhejiang University Hangzhou 310058 China; ^2^ Innovation Center in Zhejiang University State Key Laboratory of Component‐Based Chinese Medicine Hangzhou 310058 China

**Keywords:** DNA‐based barcoding, sample multiplexing, single‐cell RNA sequencing

## Abstract

Barcoding technology has greatly improved the throughput of cells and genes detected in single‐cell RNA sequencing (scRNA‐seq) studies. Recently, increasing studies have paid more attention to the use of this technology to increase the throughput of samples, as it has greatly reduced the processing time, technical batch effects, and library preparation costs, and lowered the per‐sample cost. In this review, the various DNA‐based barcoding methods for sample multiplexing are focused on, specifically, on the four major barcoding strategies. A detailed comparison of the barcoding methods is also presented, focusing on aspects such as sample/cell throughput and gene detection, and guidelines for choosing the most appropriate barcoding technique according to the personalized requirements are developed. Finally, the critical applications of sample multiplexing and technical challenges in combinatorial labeling, barcoding in vivo, and multimodal tagging at the spatially resolved resolution, as well as, the future prospects of multiplexed scRNA‐seq, for example, prioritizing and predicting the severity of coronavirus disease 2019 (COVID‐19) in patients of different gender and age are highlighted.

## Graphical abstract

DNA‐based barcoding technology enables simultaneous large‐scale sample multiplexing for single‐cell RNA sequencing (scRNA‐seq) and greatly reduces technical batch effects which make it an unparalleled performance in high‐throughput perturbation screening and tracking the dynamic process of cell differentiation. The breakthroughs of combinatorial labelling, barcoding in vivo, and multimodal barcoding at the spatially resolved resolution make more potential applications in life sciences possible.

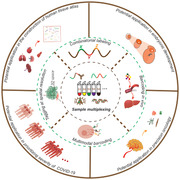

## 1 Introduction

Single‐cell RNA sequencing (scRNA‐seq) enables transcriptome‐wide expression profile of individual cells and has gained numerous developments in recent years. Transcriptional data obtained by scRNA‐seq can be used to explore cell heterogeneity,^[^ ^1^, ^2^, ^3^ ^]^ cluster cells,^[^ ^4^, ^5^, ^6^ ^]^ analyze cell–cell communication,^[^ ^7^ ^]^ and depict cell differentiation trajectories in pseudo‐time.^[^ ^8^ ^]^ Hence, scRNA‐seq is a powerful tool that is widely applied in many biological and medical fields.

Since the advent of massive parallel RNA sequencing of single cells in 2009, many new and improved scRNA‐seq methods have been developed, mainly with the goal of increasing the throughput of cell and gene detection, as comprehensively reviewed elsewhere.^[^ ^9^, ^10^ ^]^ However, the throughput of scRNA‐seq is still limiting for many critical biomedical studies and clinical applications, for example, comprehensive screens of transcriptome perturbations associated with exposure to different drugs, in specific cell lines, and in response to specific doses;^[^ ^11^ ^]^ assessments of differentially expressed genes in multiple individuals or across diverse disease stages;^[^ ^12^ ^]^ and depiction of the cell differentiation trajectory upon exposure to different stimuli.^[^ ^13^ ^]^

**Figure 1:**
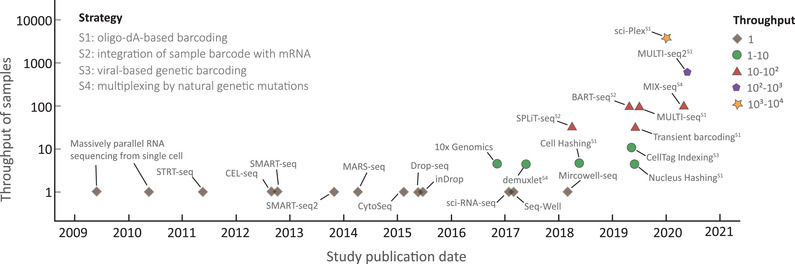
Sample throughput of representative scRNA‐seq methods. The approaches are denoted by five shapes, according to the number of samples analyzed in the study, as shown in the figure key. S1‐4 respectively represents the strategy corresponding to each barcoding approach, namely, S1: Oligo‐dA‐based barcoding; S2: Integration of sample barcode with mRNA; S3: Viral‐based genetic barcoding; S4: Multiplexing by natural genetic mutations.

Performing standard single‐cell transcriptome sequencing independently for numerous samples is unrealistic and impractical, as it is associated with excessive operation costs and reagent use, and severe batch effects. By contrast, multiplexing strategies have been widely used in various research fields, greatly increasing the number of measured parameters in a single experiment.^[^ ^14^ ^]^ Since 2017, multiplexing methods have been successively developed for simultaneous scRNA‐seq of numerous samples. These methods rely on DNA‐based barcoding that enables the pooling of all barcoded samples into a single mixed sample for analysis (**Figure** **1**). In these experiments, each sample is labeled using a unique sequential DNA barcode, where each position can be filled by one of four possible bases. This results in an enormous number of unique combinations that are read by a sequencer. The DNA barcode is becoming the most versatile label in sample multiplexing for scRNA‐seq. Accordingly, the throughput of sample multiplexing for scRNA‐seq has increased from 8 in the earliest approach, called demuxlet,^[^ ^12^ ^]^ to nearly 5000 in sci‐Plex^[^ ^11^ ^]^ (Figure 1). The current multiplexing methods with different technical designs have been successfully used in specific applications; however, a versatile technique to meet most biological researches is still lacking. Hence, a comprehensive summary and detailed comparison of the well‐established multiplexing techniques are essential for determining the appropriate method for diverse applications.

Here, we review the recent developments in barcoding methods for sample multiplexing. The scRNA‐seq methods can be classified into two categories: Full‐length transcript sequencing approaches and 3′/5′‐end profiling technologies; but we mainly summarize the sample multiplexing techniques of the latter, not only 3′‐end sequencing technologies enable larger throughput of single‐cell, but also it is compatible with the sample barcoding technology in principle. Notably, it's the first comprehensive review of scRNA‐seq sample multiplexing methods although it has gained tremendous attention since 2017. We first present four barcoding strategies: Sample labeling with a DNA barcode independent of mRNA; sample multiplexing by integration of a DNA barcode and mRNA; viral integration‐based genetic barcoding; and using naturally occurring mutations for multiplexing. Moreover, to determine the most suitable barcoding method according to custom requirement, a decision diagram is plotted based on distinct features such as sample/cell throughput and the performance of gene detection of various approaches. We then highlight two major applications of these methods, namely, high‐throughput perturbation screening and tracking the dynamic process of cell differentiation. And we also make the effort to comprehensively summarize the technical challenges in combinatorial labeling, barcoding in vivo, and multimodal tagging at a spatially resolved resolution which are essential for the development of barcoding technology. Finally, we discuss the advantages and shortcoming of the existing multiplexing methods and review the technical challenges in combinatorial barcoding, barcoding in vivo, and multimodal barcoding at the spatially resolved resolution, as well as, the potential application in constructing human cell atlas, embryonic development, the precision medicine of cancer and predicting the severity for coronavirus disease 2019 (COVID‐19) which would provide new insight to broaden the scope of application of barcoding technology in life sciences.

## 2 Tracking Multiplexed Single‐Cell RNA Sequencing Samples with DNA Barcodes

**Figure 2:**
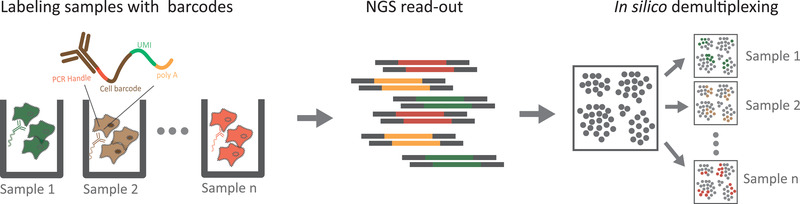
General procedures of sample multiplexing for scRNA‐seq. Cells from different samples are labeled using unique sample‐specific oligonucleotides with DNA barcodes. The samples are then pooled for simultaneous library construction and sequencing. Next, sample‐specific barcodes are read alongside single‐cell transcriptomes. This is followed by in silico demultiplexing based on a predefined barcode sequence, to assign sample identity to each cell.

In general, the barcoding flowchart for sample multiplexing consists of three steps (**Figure** **2**): labeling distinct samples with predefined or specific barcodes; calling of sample‐specific barcodes by a sequencer; and in silico demultiplexing to assign each cell to the sample of origin. The differences between the various multiplexing approaches for scRNA‐seq are mainly reflected in the different labeling methods used.

## 3 Advances in Cutting‐Edge Sample Multiplexing Methods for Single‐Cell RNA Sequencing

**Figure 3:**
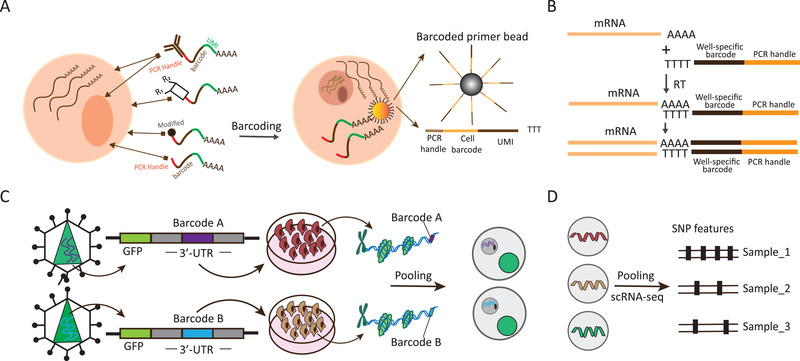
Schematic overview of four DNA‐based barcoding strategies. A) S1: Oligo‐dA‐based barcoding. For this strategy, sample‐specific DNA barcode is generally polyadenylated at the 3′‐end and is structurally similar to endogenous mRNA. It can be captured alongside mRNA by the same barcoded bead that is cell‐specific. Several direct and indirect approaches are available for assigning sample barcodes to cells, depending on sample type, for example, polyadenylated barcoded ssDNA, which diffuses into a fixed nucleus and directly labels the mRNA from nucleus; chemically modified barcode oligonucleotides, which bind with cellular proteins for cell tagging; and a transient transfection with a barcode, which labels the cell. As an indirect labeling approach, barcoded antibody and lipid‐tagged barcode are used to target ubiquitous proteins or the nucleus core complex, and the cellular membrane, accordingly, to label the cell. B) S2: Multiplexing by integration of DNA barcode with mRNA. The integrated cDNA is generated via a reverse‐transcription (RT) reaction with sample‐specific barcoded primers that are poly(dT) at the 3′‐end or have the sequences complementary to transcript. C) S3: Viral integration‐based genetic barcoding. Sample barcodes (barcode A, barcode B) and green fluorescent protein (GFP) are merged to be incorporated into genome sequence respectively via lentiviral transduction. Sample barcode can be transcribed into polyadenylated transcripts, which are efficiently captured along with endogenous transcripts in single‐cell library construction. D) S4: Exploitation of naturally occurring genetic mutations. Here, natural mutations are used as barcodes of individuals or cancer cell lines. Demultiplexing is mainly based on the dissection of SNP variation using computational tools, such as demuxlet.

Generally, as shown in **Figure** **3**, the existing strategies for sample multiplexing for scRNA‐seq with DNA‐based barcoding can be divided into the following four categories: Simultaneous capturing of the barcode and mRNA, involving direct and indirect barcoding; multiplexing based on cDNA barcoding; multiplexing based on viral integration, which introduces inheritable genetic barcode in vivo that persists over time; and multiplexing based on naturally occurring genetic mutations, which leverages genetic mutations as individual markers. Each strategy has its own merits and limitations, with a unique design and specific applications. A detailed comparison of the existing sample multiplexing methods for scRNA‐seq is presented in **Table** **1**, focusing on the differences in tagging cells from different samples, throughput, demultiplexing accuracy, and others.

**Table 1:** Detailed comparison of prospective sample multiplexing methods

Strategy	Method	Tagging cells from different samples	Throughput (sample)	Throughput (cell)	Median genes per cell	Single cell/nucleus	Cell state	Sample type	Single‐cell platform	Ref.
S1	Cell Hashing^a),*)^	Barcoded antibodies target ubiquitously expressed proteins like B2M and CD298 on cellular membrane	8‐plex	16 976	NA	Single cell	Live cell	Human PBMCs	10 × chromium^[^ ^15^ ^]^	[16]
S1	Nucleus Hashing^a),*)^	Barcoded antibodies target nucleus pore complex	8‐plex	13 578	500∼3500	Single nucleus	Frozen cell	Fresh‐frozen murine or human brain cortex	10 × chromium^[^ ^15^ ^]^	[17]
S1	Transient barcoding^a),*)^	Short barcodes with oligo‐dA are transiently transfected into cytoplasm	48‐plex	3091	≈2900	Single cell	Live cell	Cell line	Drop‐seq^[^ ^18^ ^]^	[19]
S1	MULTI‐seq^a),*)^	Lipid‐ and cholesterol‐modified barcodes are attached to membrane	96‐plex	14 377	1200–4000	Single cell and single nucleus	Live cell and nucleus	Cell line	10 × chromium^[^ ^15^ ^]^	[13]
S1	Chemically tagging^b),*)^	Methyltetrazine‐modified barcodes are covalently linked to cellular membrane	96‐plex	23 097	3540 (human); 2090 (mouse)	Single cell	Fixed cell	Cell line	Modified 10 × genomics protocol	[20]
S1	MULTI‐seq2^a),*)^	Lipid‐ and cholesterol‐modified barcodes are attached to membrane	576‐plex	58 088	3649	Single cell	Live cell	Cell line	10 × chromium^[^ ^15^ ^]^	[21]
S1	sci‐Plex^b),*)^	Unmodified barcodes with oligo‐dA specifically diffuse into nucleus	≈5000 samples	≈650 000	<2000	Single nucleus	Fixed nucleus	Cell line	Combinatorial indexing^[^ ^22^ ^]^	[11]
S2	SPLiT‐seq^a)^	Barcoded primers hybridize with mRNA via RT reaction	48‐plex	15 6049	677	Single cell and single nucleus	Fixed cell and nucleus	Mouse brain and spinal cord	Combinatorial indexing^[^ ^22^ ^]^	[23]
S2	BART‐Seq^b)^	Dual barcoded primers hybridize with target transcripts via RT reaction	96‐plex	4500	19	Single cell	Live cell	Human pluripotent stem cells	NA	[24]
S3	CellTag Indexing^a)^	Barcodes are incorporated into cell genome through lentivirus infection	10‐plex	4763	1200–1800	Single cell and single nucleus	Live cell	Cell line	10 × chromium^[^ ^15^ ^]^	[25]
S4	demuxlet^a),*)^	Natural genetic mutations serve as inherent barcodes	8‐plex	25 918	NA	Single cell	Live cell	PBMCs from lupus patients	10 × chromium^[^ ^15^ ^]^	[12]
S4	MIX‐seq^a),*)^	SNPs act as inherent barcodes	99‐plex	7317	NA	Single cell	Live cell	Cancer cell line	10 × chromium^[^ ^15^ ^]^	[26

Multiplexing strategies: S1, oligo‐dA‐based barcoding; S2, integration of a DNA barcode with mRNA; S3, genetic barcoding based on viral integration; S4, exploitation of naturally occurring genetic mutations; ^a)^ and ^b)^ correspond to 1D and 2D barcodes, respectively; ^*)^The method can be used to recognize and remove doublets; Sample throughput reflects the actual number of analyzed samples, not the theoretical value; NA represents not available in publication.

### 3.1 Oligo‐dA‐Based Barcoding

ScRNA‐seq technologies have been broadly applied in various biomedical studies with the advent of microfluidic devices and the application of barcoded beads. The bead surface anchors millions of barcoded RT primers to uniquely barcode single cells. Single cells are isolated and encapsulated into droplets with a barcoded bead, cell lysis buffer, and RT mix; after rapid cell lysis, all mRNAs from the single cell are labeled by cell‐unique identifiers present within barcoded RT primers on beads.

Several novel sample multiplexing methods for scRNA‐seq have been proposed based on a sample barcode that is structurally similar to mRNA and can be captured in parallel with mRNA by complementary hybridization on beads via the common polyadenylated tail. Their feasibility has been demonstrated. Generally, single‐strand DNA oligonucleotide, which is a specific sequence composed of 6 to 12 nucleotides, serves as the vector of sample barcode. In addition, the vector also contains a universal PCR handle sequence for amplification by PCR, and a unique molecular identifier for accurately quantifying transcript abundance by eliminating PCR amplification bias. Multiplexing methods based on the idea of an mRNA analogue acting as a sample label technically differ in the manner in which sample‐specific barcodes are attached to single cells or nuclei in different samples (Figure 3A).

Cell Hashing and Nucleus Hashing strategies were designed by Stoeckius^[^ ^16^ ^]^ and Gaublomme,^[^ ^17^ ^]^ respectively. In Cell Hashing, DNA‐barcoded antibodies targeting cellular surface proteins are designed to specifically tag cells from diverse samples, which are pooled and analyzed in a single scRNA‐seq experiment. Barcoded antibody is prepared by using inverse electron‐demand Diels–Alder reaction to covalently bind Hashtag oligonucleotide containing a 12‐bp barcode to the antibody. By sequencing sample barcode in parallel with endogenous mRNA, wherein both have been tagged with identical cell barcode during library construction, the sample identity of each cell can be mapped. This multiplexing approach has been demonstrated in an experiment comprising eight human peripheral blood mononuclear cell (PBMC) mixed sample in a simultaneous single‐droplet‐based scRNA‐seq. In contrast to Cell Hashing, Nucleus Hashing relies on barcoded antibodies to uniquely label and target the nucleus pore complex in distinct biological samples. A computational tool named “DemuxEM” was designed for in silico demultiplexing.^[^ ^17^ ^]^ It assigns each barcoded single nucleus to the original sample and identifies inter‐sample multiplets. This enables “super‐loading” on a commercial scRNA‐seq platform to lower reagent cost of library construction. This is a custom multiplexing approach for single‐nucleus (sn) RNA‐seq. It has appreciably extended the spectrum of sample specimen types available for analysis, for example, samples that are not easy to dissociate and clinical samples that had been frozen for extended periods of time.

It has been demonstrated that the “anchor,” lipid‐modified oligonucleotide (LMO) scaffolds, can rapidly and stably conjugate with the cellular membrane of a live cell via a hydrophobic 5′‐lignoceric acid amide.^[^ ^27^ ^]^ McGinnis and colleagues^[^ ^13^ ^]^ introduced LMOs into MULTI‐seq, a novel method for multiplexed scRNA‐seq or single nucleus RNA sequencing (snRNA‐seq). LMOs, which consist of lipid scaffold, a 5′‐PCR handle, an 8‐bp sample barcode, and 3′‐poly(A) sequence, localize to the cellular membrane or nuclei without influencing cell viability and endogenous gene expression pattern. Sample‐specific LMOs and endogenous mRNA within a single cell are simultaneously captured and indexed by same cell barcode during library construction. MULTI‐seq has been leveraged to dissect the dynamic transcriptional changes in T cells treated with ionomycin and phorbol 12‐myristate 13‐acetate (PMA) across eight time points.^[^ ^13^ ^]^

Shin and co‐workers^[^ ^19^ ^]^ developed a universal sample barcoding method by transiently transfecting live cells with short barcode oligonucleotides (SBOs) to respectively mark different K562 cell samples treated by 45 BCR‐ABL‐targeting drugs. SBO, a single‐stranded DNA (ssDNA) oligonucleotide, includes a sample‐specific sequence and a poly(A) sequence. The poly(A) sequence ensures that endogenous mRNAs and predefined SBOs from the same single cell can be captured in parallel and share a same cell identifier. Accordingly, the authors^[^ ^19^ ^]^ analyzed a 48‐plex drug treatment experiment in a single Drop‐seq^[^ ^18^ ^]^ run to successfully reveal specific transcriptional T‐cell responses and signatures of each drug.

It has been demonstrated that the ssDNA oligonucleotide can be located in the nucleus of a permeabilized cell by diffusing. Accordingly, sci‐Plex^[^ ^11^ ^]^ relies on labeling the nucleus with a combination of two unmodified ssDNA oligonucleotides that are polyadenylated and can be used for simultaneous combinatorial indexing of mRNA from a specific sample for scRNA‐seq. In sci‐Plex, transcriptome profiling at a single‐cell resolution is achieved through combinatorial barcoding, which only involves pipetting steps, with no need for any complex devices. Without the limitation of a microfluidic device, sci‐Plex generates millions of single cells barcoded by using unique combinatorial indices that consist of three rounds of indices, where each round contains 384 indices. Indeed, it has allowed parallel transcriptome profiling of ≈650 000 single‐cells from 4608 independent samples in a single high‐throughput screening experiment.

Another versatile scRNA‐seq sample multiplexing method involves chemical labeling of fixed cells by attaching dual unique DNA oligonucleotides called “ClickTags” to cellular proteins.^[^ ^20^ ^]^ Dual ClickTags are affixed to the proteins by Diels–Alder chemistry and the heterobifunctional amine‐reactive cross‐linker NHS‐*trans*‐cyclooctene and has been successfully applied in a 96‐plex perturbation experiment.

### 3.2 Sample Multiplexing by Merging of DNA Barcode and mRNA

Introduction of barcodes during RT is a widely used approach for labeling thousands of different transcripts. It involves the use of barcoded RT primers that enable the incorporation of barcodes into the cDNA of individual samples after RT.^[^ ^28^, ^29^ ^]^ Differently from mRNA analogue barcodes that can be captured in parallel with endogenous mRNA, and marked with the same cell identifiers by barcoded beads, a barcode for cDNA‐based multiplexing labels all mRNA species of cells from diverse samples with unique sample barcodes upon hybridization of an RT primer and mRNA. This is followed by the generation of cDNA, which contains the sample barcode and mRNA message (Figure 3B).

Split‐pool ligation‐based transcriptome sequencing (SPLiT‐seq),^[^ ^23^ ^]^ which is similar to sci‐Plex,^[^ ^11^ ^]^ enables simultaneous indexing of the cellular origin of RNA from hundreds of thousands of fixed cells or nuclei in a single RNA‐seq by combinatorial barcoding. SPLiT‐seq involves four rounds of combinatorial barcoding for indexing each transcript without microfluidic device. During the first round of barcoding for sample multiplexing, the formaldehyde‐fixed cells or nuclei are evenly suspended and distributed into a 96‐well plate, where every well represents a different biological sample. Then, cellular mRNA is tagged with well‐specific RT primers through an in‐cell RT reaction. The number of multiplexed biological samples can be scaled up to 384 by implementing the first‐round barcoding in a 384‐well plate. Undoubtedly, this approach can accelerate the widespread adoption of multiplexed scRNA‐seq. For instance, a comprehensive transcriptional analysis of 48‐plex samples of the brain and spinal cord from 11 mice two days postpartum was performed,^[^ ^23^ ^]^ resulting in the characterization of 156 049 single‐nucleus transcriptomes.

A highly sensitive, inexpensive targeted scRNA‐seq method, barcode assembly for targeted sequencing,^[^ ^21^ ^]^ has been developed for the simultaneous targeted sequencing of genome and transcriptome of large‐scale samples at either single‐cell resolution or in bulk. In BART‐Seq, 8‐mer DNA barcodes are inserted into an invariant set of forward and reverse primers during two rounds of hybridization. A complementary adapter enables the labeling of targeted transcript cohorts with dual indices. The dual indices for targeted transcript labeling are based on the incorporation of sample‐specific dual barcodes and transcripts. BART‐seq has been used to investigate the mechanisms underpinning the differentiation propensity of stem cells. In the experiment, the cells were exposed to various media that asynchronously activates Wnt/*β*‐catenin pathway. Compared to droplet‐based,^[^ ^18^ ^]^ 10 ×,^[^ ^15^ ^]^ and other scRNA‐seq methods, BART‐seq can profile a wider range of RNA species including lncRNAs in a single cell, provided specific primer sets are employed.

Finally, similar to the approaches based on the incorporation of a barcoded primer into the cDNA, some novel methods of sample multiplexing have been developed for bulk RNA‐seq, such as, DRUG‐seq^[^ ^28^ ^]^ and PLATE‐seq.^[^ ^29^ ^]^

### 3.3 Viral Integration‐Based Genetic Barcoding

A unique sample barcode can be virally integrated into the genome to act as a stable genetic barcode of the cell, because it is then transcribed into a known polyadenylated transcript that can be analyzed in parallel with the transcriptome (Figure 3C). As a complement of instant labeling multiplexing based on a transient transfection with DNA oligonucleotides, barcoded antibodies, chemically modified DNA oligonucleotides, and lipid‐tagged DNA oligonucleotides, application of predefined genetic heritable barcodes allows for cell population labeling, pooling, and tracking over time. Several methods have been coupled with CRISPR editing to generate genetic barcodes as cell tags for lineage tracing.^[^ ^30^, ^31^, ^32^, ^33^, ^34^, ^35^, ^36^, ^37^ ^]^ However, their utility for large‐scale sample multiplexing has not yet been demonstrated.

An approach known as “CellTag Indexing”^[^ ^25^ ^]^ was recently introduced for multiplexing biological samples by stable genetic barcoding using heritable DNA barcodes. The approach utilizes a special design of modified green fluorescent protein (GFP) gene where 8‐bp sample barcode named CellTag is located in its 3′‐UTR, followed by an SV40 polyadenylation signal sequence (Figure 3C). The predefined CellTag gene would be transcribed as polyadenylated transcript after effective lentiviral transduction of the assembled GFP DNA. GFP enables the direct quantification of the transduction efficiency with lentivirus carrying CellTag. CellTag Indexing gives rise to heritable barcode maintenance in a population for long‐term either in vivo or in vitro, based on which it can precisely profile the dynamic characteristics of engraftment and differentiation at single‐cell resolution. However, safety issues and ethical concerns of in vivo multiplexing technologies should be taken seriously.

### 3.4 Exploitation of Natural Genetic Mutations for Multiplexing

Natural genetic mutations, such as, single‐nucleotide polymorphisms (SNPs), result in genotype variations that can serve as unique genetic identifiers of cells originating from non‐isogenic samples. Harnessing genetic barcodes to determine the identity of each cell in genetically distinct samples enables multiplexed scRNA‐seq experiments. The genotype variations of individual samples can be characterized by multiplexed scRNA‐seq analysis (Figure 3D).

Demuxlet^[^ ^12^ ^]^ is a computational tool for demultiplexing of pooled samples and identifying doublets of cells from different samples by multiplexed scRNA‐seq–based genotyping. The method assigns each cell to the sample of origin by statistically evaluating the maximum likelihood of RNA‐seq reads that overlap a series of SNPs in a single cell. Excellent performance of demuxlet was demonstrated by an analysis of simulation data for 2–64 individuals. The tool demultiplexed 97% of singlets and identified 92% of doublets in a pool of 64 samples, with 50 SNPs analyzed in each cell. In another study, samples from eight patients with lupus were multiplexed for pooled scRNA‐seq to characterize cell type specificity and differences in the response to IFN‐*β* across individuals. Based on the naturally occurring genetic variations, the optimal number of samples for multiplexing is approximately 20, in terms of processing and doublet rates originating in the current microfluidic devices.

Several computational tools for demultiplexing have been developed that function similarly to demuxlet. One example is Vireo, a computationally efficient Bayesian model for reconstructing sample identity of each cell without a genotype reference.^[^ ^38^ ^]^ Another demultiplexing method, GMM‐Demux, based on a Gaussian mixture model^[^ ^39^ ^]^ has been developed to precisely recognize and remove multiplets in barcoding approaches. In addition, multiplexed interrogation of gene expression through scRNA‐seq (MIX‐seq),^[^ ^26^ ^]^ a sample multiplexing technique, can be used to profile the post‐perturbation response in a mixed cellular context, followed by sample demultiplexing based on SNPs. In addition, MIX‐seq coupled with cell hashing method is capable to implement transcriptional response analysis across treatment time or drug dose to identify cell line‐specific and shared signatures. Further, a computational demultiplexing method has been developed for the identification of sample origins of each cell based on the SNP profile. It can be applied to demultiplexing >500 cell‐line pools, with as few as 50–100 SNP sites detected per cell.

## 4 Guidelines for Choosing the Most Suitable Barcoding Technique

**Figure 4:**
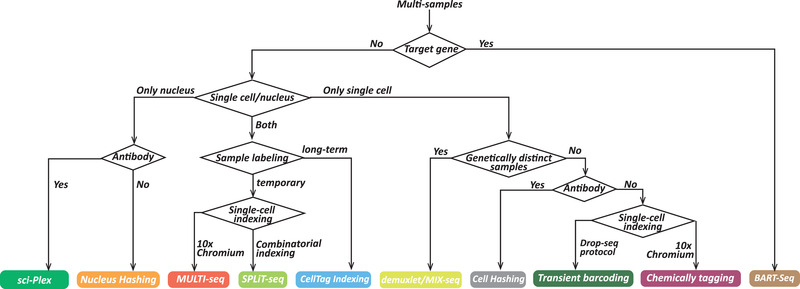
Decision diagram to help choose the most appropriate multiplexing methods according to requirement. Each technique is represented by a rectangular box with different colors.

As shown in Table 1, each method of multiplexing has different characteristics and functions, which can help us to choose a more appropriate approach to be applied in new research directly or with customized improvement according to demand. The detailed decision diagram for suitable multiplexing technique is integrated in **Figure** **4**. For example, if the command is for target transcript cohort, BART‐seq^[^ ^24^ ^]^ is a tailored choice. For a panel of druggable compounds that need to be investigated for transcriptionally distinct or common response across multiple cancer contexts in vitro, the combination of MIX‐seq^[^ ^26^ ^]^ and Cell hashing method,^[^ ^16^ ^]^ which barcodes cellular contexts and chemicals separately, would be suitable. The existing methods are complementary in many aspects. Some are aimed at the analysis of live or fixed whole cells, while others are specific for the analysis of the nucleus. snRNA‐seq technology is a good solution for complex organs like the brain, solid tissues that are difficult to dissociate, and archived tissues. Further, some multiplexing technologies have been designed for specific applications, such as MIX‐seq^[^ ^26^ ^]^—used for characterizing transcriptional variation in hundreds of cancer cell lines in response to different treatments—BART‐seq^[^ ^24^ ^]^—for accurate detection and diagnosis of some cancer mutations—and CellTag Indexing,^[^ ^25^ ^]^ which enables the tracking of cell behavior over time and can be used to observe the dynamic changes in engrafted cells in vivo. However, in terms of the number of multiplexed samples, multiplexing technologies that are based on microfluidic devices for single‐cell preparation are limited, as the number of samples is inversely proportional to the number of cells contained in each sample. However, as shown in Table 1, both sci‐Plex^[^ ^11^ ^]^ and SPLiT‐seq^[^ ^23^ ^]^ can support an increasing number of cells by eliminating the limitations associated with the use of microfluidic technology for cell suspension preparation. Additionally, these strategies employ combinatorial indexing technology and physical split‐pool operation to achieve unique labeling of nearly one million (possibly more) single cells. It is noteworthy that the gene detection performance of each sample barcoding methods, determined by both scRNA‐seq protocols and sequencing depth, is essential to learn the pros and cons of different barcoding approaches. The single‐cell libraries of these sample multiplexing methods are constructed via 10 × Chromium,^[^ ^15^ ^]^ sci‐RNA‐seq,^[^ ^22^ ^]^ and drop‐seq^[^ ^18^ ^]^ protocols whose gene detection performance have been comprehensively compared by Ding et al.^[^ ^40^ ^]^ via a benchmarking experiment across seven representative scRNA‐seq methods.

## 5 Applications of Single‐Cell RNA Sequencing Sample Multiplexing Approaches

**Figure 5:**
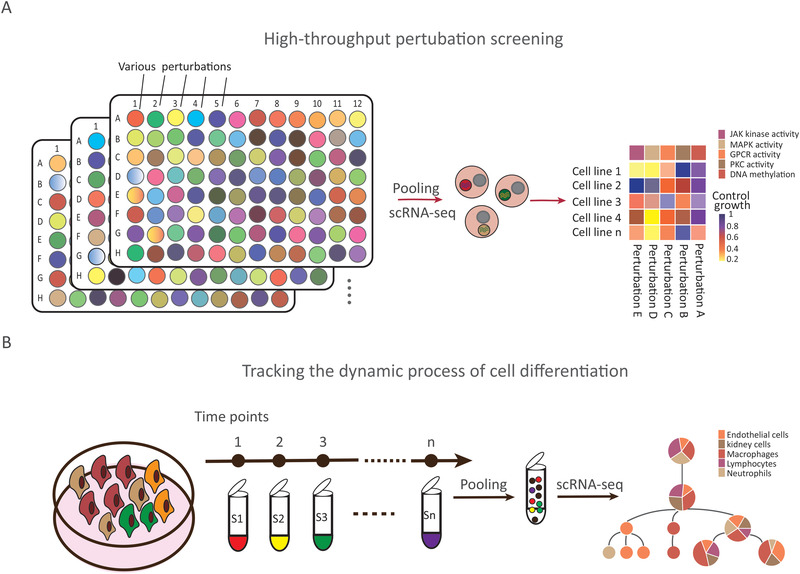
Applications of sample multiplexing technologies. Multiplexing approaches are used to address two main categories of “hot topics.” A) High‐throughput perturbation screening. B) Tracking the dynamic process of cell differentiation.

Large‐scale sample multiplexing with barcoding substantially increases the number of samples for scRNA‐seq, while at the same time facilitating library construction, lowering the reagent costs, and reducing batch effects. Sample multiplexing strategy can hence provide great insight for high‐throughput perturbation screening and tracking the dynamic process of cell differentiation (**Figure** **5**).

### 5.1 High‐throughput Perturbation Screening

The global transcriptome expression profiling—serving as a high‐content phenotypic readout—has been universally used in high‐throughput screens (HTS) of chemicals.^[^ ^28^, ^29^, ^41^ ^]^ This overcomes the limitations associated with most conventional HTS whose output is simple or superficial, such as, cell viability,^[^ ^42^, ^43^ ^]^ morphology,^[^ ^44^ ^]^ or reliance on a few exogenous reporters.^[^ ^45^ ^]^

DRUG‐seq^[^ ^28^ ^]^ is a powerful tool that ultra‐performs in HTS by enabling the profiling of transcriptomes in response to 433 compounds across eight doses by multiplexed bulk RNA‐seq, to group the chemicals into functional clusters based on the respective mechanism of action. However, the heterogeneous response of transcriptionally distinct cell subpopulations cannot be characterized using this approach. Further, the per‐sample cost of standard scRNA‐seq remains high, which greatly restricts the sample size of screens at single‐cell resolution.

ScRNA‐seq sample multiplexing methods enable scalable parallel screening of whole‐transcriptome profiles of single cells for diverse perturbations (Figure 5A). sci‐Plex^[^ ^11^ ^]^ has been developed to scale up HTS. Specifically, it was used to profile thousands of individual perturbations elicited by 188 chemicals including enzyme‐targeted compounds in three cancer lines, with four doses, and two replicates. Half of the analyzed chemicals were found to target transcriptional and epigenetic regulators. Specific transcriptional responses of cell lines to each chemical were thus readily characterized and the mechanisms of action of histone deacetylase inhibitors were demonstrated, that is, cell‐cycle arrest and effect on acetyl‐CoA metabolism. As another example, MULTI‐seq^[^ ^13^ ^]^ has been leveraged to capture the dynamics of T‐cell activation in a 96‐plex perturbation experiment involving primary human mammary epithelial cells, to investigate transcriptional responses to various combinations of signaling molecules. By using the same approach, a 576‐plex scRNA‐seq experiment^[^ ^21^ ^]^ was performed to screen the context specificity of epithelial–mesenchymal transition by simultaneous expression profiling of various samples, with three inducing factors, four different cancer cell lines, and 12 distinct time‐course experiments.

Further, kinase inhibitor screens were implemented to identify signaling dependencies of diverse epithelial–mesenchymal transition responses in distinct cell lines exposed to different kinase inhibitors and inducers, with up to 384 combinations. For instance, Gehring and colleagues^[^ ^20^ ^]^ analyzed a 96‐plex array comprising combinations of various concentrations of six growth factors by multiplexing 96 scRNA‐seq samples of live mouse neural stem cells plated in a 96‐well plate. The authors chemically tagged cellular proteins with DNA barcodes and revealed a complex interplay between the perturbants. Further, Shin et al.^[^ ^19^ ^]^ analyzed a 48‐plex drug array of 45 target inhibitors and three DMSO controls in K562 cell line. They barcoded the samples by transient transfection with SBO to characterize drug‐specific transcriptional responses at single‐cell level and evaluated cell toxicity of each drug by cell counting. A comprehensive analysis of the drug screen by unsupervised clustering revealed responsive heterogeneity of a distinct cell subpopulation treated with diverse drugs.

### 5.2 Tracking the Dynamic Process of Cell Differentiation

Unlike traditional single‐cell data obtained in response to a single stimulus or at a single time point, followed by pseudo‐time analysis to infer the cell differentiation trajectory, sample multiplexing can be used for simultaneous scRNA‐seq of samples, with multiple differentiation stimuli and multiple time points. This allows characterization of cell transcription profiles to track the differentiation trajectory of cells much more accurately and in detail than what is possible using conventional methods (Figure 5B). For instance, CellTag Indexing^[^ ^25^ ^]^ was used to track cell engraftment and differentiation in vivo over 7 weeks. CellTags were used to label Ecad^High^ and Ecad^Low^ induced endoderm progenitors. Further, McGinnis and colleagues leveraged MULTI‐seq^[^ ^13^ ^]^ to track T‐cell differentiation dynamics by multiplexing Jurkat cells treated with ionomycin and PMA across eight time points. In another study, SPLiT‐seq^[^ ^23^ ^]^ was utilized to tracking neuronal differentiation trajectories within the cerebellum by transcriptional profiling of ≈156 K single‐nucleus from 11 specimens. The experiment identified two types of Purkinje cells with specific pattern of gene expression. Further, progenitor cells were demonstrated to be able to either differentiate into stellate/basket cells or Golgi cells.

## 6 Technical Challenges

### 6.1 Combinatorial Barcoding through Multiple Hybridization

**Figure 6:**
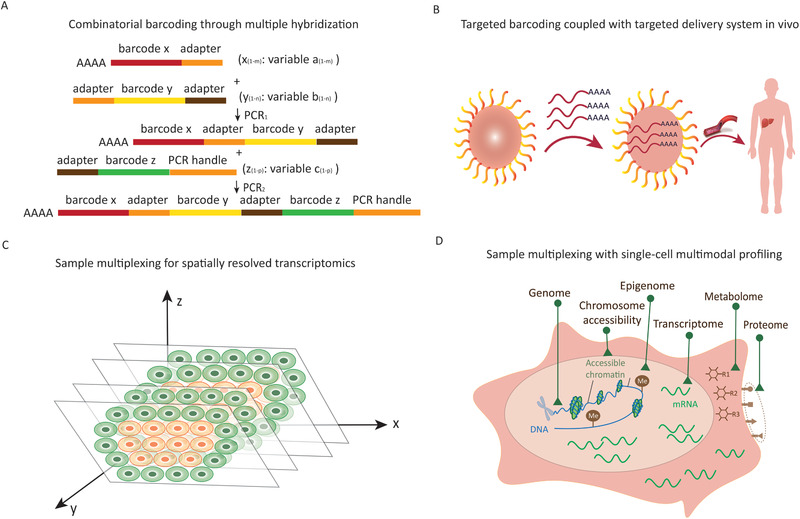
Critical technical challenges of barcoding technology for sample multiplexing. A) Combinatorial barcoding through multiple hybridization. B) Targeted barcoding coupled with targeted delivery system in vivo. C) Large‐scale sample multiplexing with single‐cell transcriptome profiling, alongside determination of a cellular spatial position, for establishing a complete 3‐dimensional (3D) spatial transcriptome atlas representative of many individuals. D) Sample multiplexing with multiple‐modality profiling within the same single cell to deepen our understanding of the function and composition of complex tissues, extending the range of biomedical applications.

Currently, the main means of greatly increasing sample throughput involve the use of synthetic barcodes. However, multiplexing methods where one barcode is used to only label one sample, such as SPLiT‐seq,^[^ ^23^ ^]^ MULTI‐seq,^[^ ^13^ ^]^ transient barcoding,^[^ ^19^ ^]^ and hashing,^[^ ^16^, ^17^ ^]^ are not economical, user‐unfriendly and limit the sample capacity of multiplexing. The use of multi‐dimensional barcodes can effectively compress the barcode space. For example, in sci‐Plex,^[^ ^11^ ^]^ the use of 96 and 52 barcodes for each well and each plate respectively in a 96‐well plate setup results in the generation of 4992 combinations of barcodes. However, in sci‐Plex, the 2D barcodes have to be assigned to nuclei simultaneously. It is essential to develop a novel combinatorial barcoding method through multiple hybridization by PCR with multi‐dimensional barcodes, so that it can be used to barcode samples for tracking various time‐course as well as larger barcode combination. As shown in **Figure** **6**A, first dimension barcodes conjugate mRNA via the hybridization of poly‐A and poly‐T during RT reaction. All dimension barcodes are merged into a combinatorial barcode after two rounds of PCR hybridization of adapters. Each barcode dimension can represent an information dimension, such as, the disease stage, tumor type, or even the geographical location. This greatly expands the application of sample multiplexing technologies.

### 6.2 Targeted Barcoding In Vivo

With the development and maturation of multi‐sample scRNA‐seq technology, many biological science questions that had been previously deemed challenging have been successfully addressed, for example, by high‐throughput perturbation screening and tracking the dynamics of cell differentiation. However, barcoding in vivo remains confined to the labeling of cells in a single in vivo sample. In combination with CRISPR genomic editing, it is widely used to follow the development and differentiation of cells or embryos, and to predict cell lineage and fate.^[^ ^31^, ^32^, ^34^, ^35^, ^36^, ^46^ ^]^ Nonetheless, the current scRNA‐seq technology for large‐scale sample analysis is mainly applied at the in vitro level in labeled cell lines. In one approach, the barcode is integrated into the genome of a cell to be transplanted via lentiviral transfection for stable and heritable labeling. The labeled cells are then transplanted into a mouse to track them and their differentiation status.^[^ ^25^ ^]^ In another approach, based on natural mutations as a sample self‐labeling method,^[^ ^12^ ^]^ the same tissues from 10–20 samples can be mixed and pooled for a single analysis. However, the two indirect in vivo labeling technologies still have some limitations when labeling specific tissues in adult organisms and transplant is harmful to the body. Notably, with the continuous development of targeted delivery technologies,^[^ ^47^ ^]^ the targeted delivery function can be combined with stable and heritable barcode generated by CRISPR editing system for targeted tissue labeling (Figure 6B). In vivo labeling of multiple samples for parallel sequencing will greatly broaden and enrich biological research, although the technical problems associated with in vivo labeling of multiple samples, including the effect of labeling on cells in vivo, off‐target effects of markers and safety issues and ethical concerns, cannot be ignored.

### 6.3 Adding Spatial Dimension to Multiplexing

The tremendous recent advances in scRNA‐seq and techniques for spatially‐resolved transcriptomics allow simultaneous profiling of single‐cell position and transcriptome.^[^ ^48^, ^49^, ^50^, ^51^, ^52^ ^]^ In the future, as forecasted in our recent review,^[^ ^10^ ^]^ uncovering the spatial heterogeneity of an organ at single‐cell resolution will potentially allow improved mapping of the 3D transcriptional atlas of organs^[^ ^53^, ^54^, ^55^, ^56^ ^]^ (Figure 6C). Sample multiplexing for simultaneous single‐cell transcriptome profiling and decoding spatial position of a single cell across a time‐course will accelerate the construction of 4‐dimensional (4D) human single‐cell atlas by using multi‐dimensional barcodes separately.

### 6.4 Sample Multiplexing for Single‐Cell Multimodal Profiling

With the evolving technology of single‐cell genomics, it will be more accurate, sensitive, and less biased to characterize cell state and elucidate cell trajectory and function by single‐cell multimodal omics (scMulti‐omics) technologies that include single‐cell sequencing of methylome, accessible‐chromatin, genome, transcriptome, proteome, gene perturbation screening, and spatial barcoding^[^ ^57^, ^58^, ^59^, ^60^, ^61^, ^62^ ^]^ (Figure 6D). ScMulti‐omics, where the data of each omics can be mutually corroborated and supplemented, can be used for a comprehensive exploration and identification of cell characteristics.^[^ ^63^ ^]^

The current multi‐omics methods generally involve the integration of transcriptome sequencing, which serves as a mediator, and other omics technologies. Based on the barcoded antibody approach, CITE‐seq^[^ ^64^ ^]^ and REAP‐seq^[^ ^65^ ^]^ enable profiling of RNA expression and proteins at single‐cell resolution. Further, simultaneous profiling of RNA expression, protein abundance, T‐cell receptor, and cell perturbations can be achieved with ECCITE‐Seq.^[^ ^66^ ^]^ There is no doubt that the merger between sample multiplexing and single‐cell multi‐modal sequencing will break through the existing biotechnology bottleneck to substantially broaden the biomedical application range, such as, 1) identifying the subtle differences in human immune system to invasive pathogens such as viruses and the diversity of immune responses caused by allergens in different individuals, 2) enabling a more comprehensive prediction of cell behavior and identity across various experimental conditions and individuals.

## 7 Potential Future Applications

### 7.1 Construction of the Human Tissue Atlas

Rapid advances in scRNA‐seq technologies have enabled the use of single‐cell transcriptional profiling for exploring cellular heterogeneity within complex tissues or organs.^[^ ^67^, ^68^, ^69^ ^]^ In 2017, the Human Cell Atlas Project^[^ ^70^ ^]^ was initiated to provide a 3D map of different types of cells that make up human tissues, revealing how all systems are connected, and the relationship and transcriptional changes in health and disease. When completed, HCA will improve the understanding, diagnosis, and treatment of diseases.

As part of the HCA effort, Guo and colleagues^[^ ^71^ ^]^ used microwell‐seq to construct the human cell atlas with broad range of both adult and fetal tissues and specifically clarify diverse cell types within all major organs in humans. Further, Baumert and coworkers^[^ ^72^ ^]^ performed mCEL‐Seq^[^ ^73^ ^]^ to establish a human liver cell atlas, which contains approximately 10000 cells from nine normal human donors. The liver atlas is a predominant reference for the severity inference and therapy of liver diseases and will give rise to the advance of urgently needed liver models. Likewise, complete characterization of cardiac morphogenesis entails a detailed investigation of the profile and pattern of gene expression within whole organs. Accordingly, a spatiotemporal atlas^[^ ^74^ ^]^ has been developed, to systematically describe spatially resolved cellular heterogeneity in the developing human heart over three post‐conception stages. The atlas was generated by carrying out both spatial transcriptomics (ST)^[^ ^75^, ^76^ ^]^ and in situ sequencing^[^ ^77^ ^]^ with subcellular‐targeted accuracy. However, the above‐mentioned studies are limited by insufficient cell sequencing depth and a small number of clinical samples used, which undoubtedly hampers the generation of a large‐scale and highly detailed map of the human tissues at a single‐cell level.

**Figure 7:**
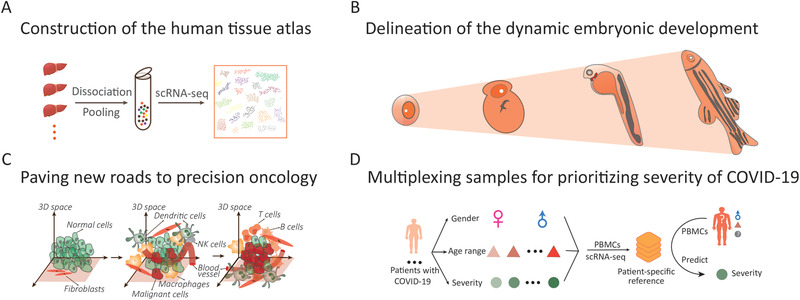
Potential applications of sample multiplexing for scRNA‐seq. A) Construction of the human tissue atlas. B) Delineation of the dynamic embryonic development. C) Paving new roads to precision oncology. D) Multiplexing samples for prioritizing severity of COVID‐19 at single‐cell resolution.

Transcriptome sequencing technologies based on combinatorial labeling of single cells for single‐cell resolution, such as SPLiT‐seq^[^ ^23^ ^]^ and sci‐Plex,^[^ ^11^ ^]^ are not dependent on microfluidic devices and can be used to analyze millions of single cells in multiple samples. By generating a knowledge database, the sample multiplexing technology meets the needs of multi‐sample parallel operations for human tissue map construction (**Figure** **7**A). Of note, human clinical samples are often cryopreserved or paraffin embedded. Sequencing technologies for single‐cell nuclear transcriptomes from multiple samples, such as, Nucleus Hashing,^[^ ^17^ ^]^ can be used to efficiently and simultaneously characterize the transcriptome profiles of numerous nuclei from single cells. These technological developments will undoubtedly contribute to the completion of the international HCA.

### 7.2 Delineation of the Dynamic Embryonic Development

Detailed description of embryonic development is an important research focus in developmental biology. Mapping its dynamics will allow cataloging of the differentiation of embryonic stem cells and facilitate the understanding of embryonic development. Single‐cell transcriptomic sequencing technology can be used to accurately depict the cellular state by providing the complete transcriptomic information for a single cell; identifying and classifying cell types in complex tissues; analyzing the cell composition of tissue; and revealing cell differentiation in health and disease. This information provides an important technical support for the study of embryonic development at single‐cell resolution. Conventionally, the most common approach for studying embryonic development involves simulation of development from single‐cell transcriptome maps of embryos at different developmental stages. However, there are some limitations to this approach. Because of the considerable cost and processing time, the number of embryo samples analyzed is small, and does not satisfactorily cover the entire developmental process in sufficient detail. In addition, the batch effect associated with sequential processing of multiple replicates undoubtedly reduce the accuracy of mapping and increases the complexity of data processing. Parallel transcriptome sequencing of multiple samples based on DNA barcoding is expected to provide a more refined and accurate picture of the dynamic embryonic development (Figure 7B). The SPLiT‐seq^[^ ^23^ ^]^ and sci‐Plex^[^ ^11^ ^]^ technologies based on composite index analyses can be used to increase the number of samples and cells in a single run, with the possibility of mapping the dynamic embryonic development.

### 7.3 Paving New Roads to Precise Oncology

Tumor is a highly complex multi‐cellular ecosystem that is mainly composed of diverse heterogeneous tumor cells, immune cells, interstitial cells, and blood vessels. Thus, precision cancer therapy is challenging due to intra‐tumoral heterogeneity and the dynamic intricate tumor microenvironment (TME), which plays a critical role in the initiation, progression, and metastasis of cancer cells. Currently, scRNA‐seq has emerged as a powerful tool to dissect the cell‐type composition within tumor and interrogate the specific cell interaction among tumor cells, immune cells, and other stromal cells (Figure 7C), which provide insight into the pathology and molecular mechanism underlying tumor immune evasion and drug resistance. By leveraging scRNA‐seq approach, single‐cell transcriptional profiles including cancer cells, stromal cells, and endothelial cells from 19 patients were generated to explore the cell state variability within melanoma tumors across distinct individuals.^[^ ^77^ ^]^ Chung et.al^[^ ^78^ ^]^ have gained 515 single‐cell transcriptome profiles from 11 primary breast tumor to distinguish non‐cancer cells from tumor cells based on copy number variations. Puram and colleagues^[^ ^79^ ^]^ have characterized the dynamic balance of distinct carcinoma cells, stromal, and immune cells in the TME and its response to immunotherapy by exploring the single‐cell transcriptomes from 18 patients with head and neck squamous cell carcinoma. Moreover, Cheng et.al.^[^ ^80^ ^]^ focused their attention on tumor‐infiltrating myeloid cells (TIMs), which are critical regulators of tumor progression; they performed a pan‐cancer analysis at single‐cell resolution by charting the transcriptional atlas where single‐cells were derived from 210 individual patients across 15 cancer types, and identified the cancer‐type‐specific expression pattern and molecular signatures of TIM. With limited sample size, more replicates and factors like spatial position, TME dynamics, gender, and age cannot be wholly included for experimental design. Therefore, it is not surprising that scaling the sample size up through barcoding method will provide more comprehensive and detailed insight into the developmental communication between tumor cells and immune cells. Additionally, this will help determine novel biomarkers for diagnosis and establish the abundant resource of single‐cell transcriptional landscape within tumor for precise prognosis and individualized therapy.

### 7.4 Multiplexing Samples for Prioritizing Severity of Coronavirus Disease 2019 at Single‐Cell Resolution

There is an urgent need to perform large‐scale detection and evaluate the severity of patients with COVID‐19, the acute pandemic caused by SARS‐CoV‐2. Multiplexing method has the potential to detect viral nucleic acids from population‐scale samples in parallel to impede the extension of the epidemic by multi‐dimensional barcoding, where the 3D barcode X, Y, and Z could be used to respectively encode the geographic message to realize the tracking of infected individuals. Moreover, investigating the immune response by characterizing transcriptional changes in PBMCs can provide insight into the pathogenesis underlying COVID‐19.^[^ ^81^, ^82^, ^83^ ^]^ Multiplexed scRNA‐seq for PBMCs from patients with COVID‐19 with varying disease severities, gender, and age can contribute toward constructing a specific transcriptional reference followed by features extraction, which can serve to identify the disease stage and predict the tropism of disease development for the samples tested (Figure 7D). This will help to develop optimal therapeutic approach for a number of patients simultaneously. The idea of multiplexing for characterizing disease state at single‐cell resolution can also be applied in other infectious diseases, such as Ebola virus, which is highlighted by the composition of cell types, cell‐specific differentiation, cell–cell interaction, and characteristic molecular markers at distinct disease stages, which are significant for precision medicine.

## 8 Concluding Remarks

Sample multiplexing strategies for scRNA‐seq have contributed to remarkable advances in biological research, for example, large‐scale screening or detection of transcriptional response to various conditions including genetic perturbations, contexts, chemicals or growth factors across doses and time at single‐cell resolution. However, the current methods for multiplexing are limited by the cost and processes of substantial barcoded primers; combinatorial indexing for tagging samples with multi‐dimensional barcodes will considerately reduce this limitation. Moreover, technical improvements in single‐cell indexing within library construction, and the reduction of sequencing costs will help address the limitation of few single‐cells per sample or narrow sequencing coverage for multiplexed samples. It will also considerably expand the range of biological research and provide improved insights for biomedicine and biotherapeutics. Further, each sample multiplexing strategy has its own assumptions and limitations, and it is therefore critical to leverage the strengths of various methods together. Sample multiplexing still has many technical and methodological challenges. Multiplexing samples for spatially resolved transcriptomics and multi‐omics sequencing will lead to it becoming an even more powerful tool for constructing 3D or even 4D human tissue atlas and characterizing the mechanisms of complicated biological phenomenon. Sample multiplexed scRNA‐seq methods will also contribute to the study of tumor biology where the cell‐type composition and interaction among cells vary at temporal and spatial resolution. Currently, sample multiplexing technology can exactly meet the needs of large‐scale rapid detection, characterize, and predict the severity of disease like COVID‐19 for a large number of individuals varying gender and age in parallel.

## Conflict of Interest

The authors declare no conflict of interest.

## References

[1] A. Takeda, M. Hollmén, D. Dermadi, J. Pan, K. F. Brulois, R. Kaukonen, T. Lönnberg, P. Boström, I. Koskivuo, H. Irjala, M. Miyasaka, M. Salmi, E. C. Butcher, S. Jalkanen, Immunity 2019, 51, 561. 31402260 10.1016/j.immuni.2019.06.027

[2] H. Li, E. T. Courtois, D. Sengupta, Y. Tan, K. H. Chen, J. Goh, S. L. Kong, C. Chua, L. K. Hon, W. S. Tan, M. Wong, P. J. Choi, L. Wee, A. M. Hillmer, I. B. Tan, P. Robson, S. Prabhakar, Nat. Genet. 2017, 49, 708. 28319088 10.1038/ng.3818

[3] E. Papalexi, R. Satija, Nat. Rev. Immunol. 2018, 18, 35. 28787399 10.1038/nri.2017.76

[4] S. Aibar, C. B. González‐Blas, T. Moerman, V. A. Huynh‐Thu, H. Imrichova, G. Hulselmans, F. Rambow, J. C. Marine, P. Geurts, J. Aerts, J. van den Oord, Z. K. Atak, J. Wouters, S. Aerts, Nat. Methods 2017, 14, 1083. 28991892 10.1038/nmeth.4463 PMC5937676

[5] H. Heaton, A. M. Talman, A. Knights, M. Imaz, D. J. Gaffney, R. Durbin, M. Hemberg, M. Lawniczak, Nat. Methods 2020, 17, 615. 32366989 10.1038/s41592-020-0820-1 PMC7617080

[6] H. Li, F. Horns, B. Wu, Q. Xie, J. Li, T. Li, D. J. Luginbuhl, S. R. Quake, L. Luo, Cell 2017, 171, 1206. 29149607 10.1016/j.cell.2017.10.019 PMC6095479

[7] X. Shao, X. Lu, J. Liao, H. Chen, X. Fan, Protein Cell 2020, 11, 866. 32435978 10.1007/s13238-020-00727-5 PMC7719148

[8] S. Zhong, S. Zhang, X. Fan, Q. Wu, L. Yan, J. Dong, H. Zhang, L. Li, L. Sun, N. Pan, X. Xu, F. Tang, J. Zhang, J. Qiao, X. Wang, Nature 2018, 555, 524. 29539641 10.1038/nature25980

[9] V. Svensson, R. Vento‐Tormo, S. A. Teichmann, Nat. Protoc. 2018, 13, 599. 29494575 10.1038/nprot.2017.149

[10] J. Liao, X. Lu, X. Shao, L. Zhu, X. Fan, Trends Biotechnol. 2020, 39, 43. 32505359 10.1016/j.tibtech.2020.05.006

[11] S. R. Srivatsan, J. L. Mcfaline‐Figueroa, V. Ramani, L. Saunders, J. Cao, J. Packer, H. A. Pliner, D. L. Jackson, R. M. Daza, L. Christiansen, F. Zhang, F. Steemers, J. Shendure, C. Trapnell, Science 2020, 367, 45. 31806696 10.1126/science.aax6234 PMC7289078

[12] H. M. Kang, M. Subramaniam, S. Targ, M. Nguyen, L. Maliskova, E. Mccarthy, E. Wan, S. Wong, L. Byrnes, C. M. Lanata, R. E. Gate, S. Mostafavi, A. Marson, N. Zaitlen, L. A. Criswell, C. J. Ye, Nat. Biotechnol. 2018, 36, 89. 29227470 10.1038/nbt.4042 PMC5784859

[13] C. S. Mcginnis, D. M. Patterson, J. Winkler, D. N. Conrad, M. Y. Hein, V. Srivastava, J. L. Hu, L. M. Murrow, J. S. Weissman, Z. Werb, E. D. Chow, Z. J. Gartner, Nat. Methods 2019, 16, 619. 31209384 10.1038/s41592-019-0433-8 PMC6837808

[14] L. Binan, E. A. Drobetsky, S. Costantino, SLAS Technol. 2019, 24, 298. 30707854 10.1177/2472630318824337

[15] G. X. Zheng, J. M. Terry, P. Belgrader, P. Ryvkin, Z. W. Bent, R. Wilson, S. B. Ziraldo, T. D. Wheeler, G. P. Mcdermott, J. Zhu, M. T. Gregory, J. Shuga, L. Montesclaros, J. G. Underwood, D. A. Masquelier, S. Y. Nishimura, M. Schnall‐Levin, P. W. Wyatt, C. M. Hindson, R. Bharadwaj, A. Wong, K. D. Ness, L. W. Beppu, H. J. Deeg, C. Mcfarland, K. R. Loeb, W. J. Valente, N. G. Ericson, E. A. Stevens, J. P. Radich, et al., Nat. Commun. 2017, 8, 14049. 28091601 10.1038/ncomms14049 PMC5241818

[16] M. Stoeckius, S. Zheng, B. Houck‐Loomis, S. Hao, B. Z. Yeung, W. R. Mauck, P. Smibert, R. Satija, Genome Biol. 2018, 19, 224. 30567574 10.1186/s13059-018-1603-1 PMC6300015

[17] J. T. Gaublomme, B. Li, C. Mccabe, A. Knecht, Y. Yang, E. Drokhlyansky, N. Van Wittenberghe, J. Waldman, D. Dionne, L. Nguyen, P. L. De Jager, B. Yeung, X. Zhao, N. Habib, O. Rozenblatt‐Rosen, A. Regev, Nat. Commun. 2019, 10, 2907. 31266958 10.1038/s41467-019-10756-2 PMC6606589

[18] E. Z. Macosko, A. Basu, R. Satija, J. Nemesh, K. Shekhar, M. Goldman, I. Tirosh, A. R. Bialas, N. Kamitaki, E. M. Martersteck, J. J. Trombetta, D. A. Weitz, J. R. Sanes, A. K. Shalek, A. Regev, S. A. Mccarroll, Cell 2015, 161, 1202. 26000488 10.1016/j.cell.2015.05.002 PMC4481139

[19] D. Shin, W. Lee, J. H. Lee, D. Bang, Sci. Adv. 2019, 5, eaav2249. 31106268 10.1126/sciadv.aav2249 PMC6520024

[20] J. Gehring, P. J. Hwee, S. Chen, M. Thomson, L. Pachter, Nat. Biotechnol. 2020, 38, 35. 31873215 10.1038/s41587-019-0372-z

[21] D. P. Cook, B. C. Vanderhyden, Nat. Commun. 2020, 11, 2142. 32358524 10.1038/s41467-020-16066-2 PMC7195456

[22] J. Cao, J. S. Packer, V. Ramani, D. A. Cusanovich, C. Huynh, R. Daza, X. Qiu, C. Lee, S. N. Furlan, F. J. Steemers, A. Adey, R. H. Waterston, C. Trapnell, J. Shendure, Science 2017, 357, 661. 28818938 10.1126/science.aam8940 PMC5894354

[23] A. B. Rosenberg, C. M. Roco, R. A. Muscat, A. Kuchina, P. Sample, Z. Yao, L. T. Graybuck, D. J. Peeler, S. Mukherjee, W. Chen, S. H. Pun, D. L. Sellers, B. Tasic, G. Seelig, Science 2018, 360, 176. 29545511 10.1126/science.aam8999 PMC7643870

[24] F. Uzbas, F. Opperer, C. Sönmezer, D. Shaposhnikov, S. Sass, C. Krendl, P. Angerer, F. J. Theis, N. S. Mueller, M. Drukker, Genome Biol. 2019, 20, 155. 31387612 10.1186/s13059-019-1748-6 PMC6683345

[25] C. Guo, W. Kong, K. Kamimoto, G. C. Rivera‐Gonzalez, X. Yang, Y. Kirita, S. A. Morris, Genome Biol. 2019, 20, 90. 31072405 10.1186/s13059-019-1699-y PMC6509836

[26] J. M. Mcfarland, B. R. Paolella, A. Warren, K. Geiger‐Schuller, T. Shibue, M. Rothberg, O. Kuksenko, W. N. Colgan, A. Jones, E. Chambers, D. Dionne, S. Bender, B. M. Wolpin, M. Ghandi, I. Tirosh, O. Rozenblatt‐Rosen, J. A. Roth, T. R. Golub, A. Regev, A. J. Aguirre, F. Vazquez, A. Tsherniak, Nat. Commun. 2020, 11, 4296. 32855387 10.1038/s41467-020-17440-w PMC7453022

[27] R. J. Weber, S. I. Liang, N. S. Selden, T. A. Desai, Z. J. Gartner, Biomacromolecules 2014, 15, 4621. 25325667 10.1021/bm501467h PMC4261982

[28] C. Ye, D. J. Ho, M. Neri, C. Yang, T. Kulkarni, R. Randhawa, M. Henault, N. Mostacci, P. Farmer, S. Renner, R. Ihry, L. Mansur, C. G. Keller, G. Mcallister, M. Hild, J. Jenkins, A. Kaykas, Nat. Commun. 2018, 9, 4307. 30333485 10.1038/s41467-018-06500-x PMC6192987

[29] E. C. Bush, F. Ray, M. J. Alvarez, R. Realubit, H. Li, C. Karan, A. Califano, P. A. Sims, Nat. Commun. 2017, 8, 105. 28740083 10.1038/s41467-017-00136-z PMC5524642

[30] K. R. Roy, J. D. Smith, S. C. Vonesch, G. Lin, C. S. Tu, A. R. Lederer, A. Chu, S. Suresh, M. Nguyen, J. Horecka, A. Tripathi, W. T. Burnett, M. A. Morgan, J. Schulz, K. M. Orsley, W. Wei, R. S. Aiyar, R. W. Davis, V. A. Bankaitis, J. E. Haber, M. L. Salit, O. R. St, L. M. Steinmetz, Nat. Biotechnol. 2018, 36, 512. 29734294 10.1038/nbt.4137 PMC5990450

[31] B. Spanjaard, B. Hu, N. Mitic, P. Olivares‐Chauvet, S. Janjuha, N. Ninov, J. P. Junker, Nat. Biotechnol. 2018, 36, 469. 29644996 10.1038/nbt.4124 PMC5942543

[32] S. Bowling, D. Sritharan, F. G. Osorio, M. Nguyen, P. Cheung, A. Rodriguez‐Fraticelli, S. Patel, W. C. Yuan, Y. Fujiwara, B. E. Li, S. H. Orkin, S. Hormoz, F. D. Camargo, Cell 2020, 181, 1410. 32413320 10.1016/j.cell.2020.04.048 PMC7529102

[33] A. Mckenna, G. M. Findlay, J. A. Gagnon, M. S. Horwitz, A. F. Schier, J. Shendure, Science 2016, 353, aaf7907. 27229144 10.1126/science.aaf7907 PMC4967023

[34] R. Kalhor, K. Kalhor, L. Mejia, K. Leeper, A. Graveline, P. Mali, G. M. Church, Science 2018, 361, eaat9804. 30093604 10.1126/science.aat9804 PMC6139672

[35] A. Alemany, M. Florescu, C. S. Baron, J. Peterson‐Maduro, A. van Oudenaarden, Nature 2018, 556, 108. 29590089 10.1038/nature25969

[36] W. Pei, T. B. Feyerabend, J. Rössler, X. Wang, D. Postrach, K. Busch, I. Rode, K. Klapproth, N. Dietlein, C. Quedenau, W. Chen, S. Sauer, S. Wolf, T. Höfer, H. R. Rodewald, Nature 2017, 548, 456. 28813413 10.1038/nature23653 PMC5905670

[37] S. O. Halperin, C. J. Tou, E. B. Wong, C. Modavi, D. V. Schaffer, J. E. Dueber, Nature 2018, 560, 248. 30069054 10.1038/s41586-018-0384-8

[38] Y. Huang, D. J. Mccarthy, O. Stegle, Genome Biol. 2019, 20, 273. 31836005 10.1186/s13059-019-1865-2 PMC6909514

[39] H. Xin, Q. Lian, Y. Jiang, J. Luo, X. Wang, C. Erb, Z. Xu, X. Zhang, E. Heidrich‐O'Hare, Q. Yan, R. H. Duerr, K. Chen, W. Chen, Genome Biol. 2020, 21, 188. 32731885 10.1186/s13059-020-02084-2 PMC7393741

[40] J. Ding, X. Adiconis, S. K. Simmons, M. S. Kowalczyk, C. C. Hession, N. D. Marjanovic, T. K. Hughes, M. H. Wadsworth, T. Burks, L. T. Nguyen, J. Kwon, B. Barak, W. Ge, A. J. Kedaigle, S. Carroll, S. Li, N. Hacohen, O. Rozenblatt‐Rosen, A. K. Shalek, A. C. Villani, A. Regev, J. Z. Levin, Nat. Biotechnol. 2020, 38, 737. 32341560 10.1038/s41587-020-0465-8 PMC7289686

[41] A. Subramanian, R. Narayan, S. M. Corsello, D. D. Peck, T. E. Natoli, X. Lu, J. Gould, J. F. Davis, A. A. Tubelli, J. K. Asiedu, D. L. Lahr, J. E. Hirschman, Z. Liu, M. Donahue, B. Julian, M. Khan, D. Wadden, I. C. Smith, D. Lam, A. Liberzon, C. Toder, M. Bagul, M. Orzechowski, O. M. Enache, F. Piccioni, S. A. Johnson, N. J. Lyons, A. H. Berger, A. F. Shamji, A. N. Brooks, et al., Cell 2017, 171, 1437. 29195078 10.1016/j.cell.2017.10.049 PMC5990023

[42] D. Shum, C. Radu, E. Kim, M. Cajuste, Y. Shao, V. E. Seshan, H. Djaballah, J. Enzyme Inhib. Med. Chem. 2008, 23, 931. 18608772 10.1080/14756360701810082 PMC3710589

[43] C. Yu, A. M. Mannan, G. M. Yvone, K. N. Ross, Y. L. Zhang, M. A. Marton, B. R. Taylor, A. Crenshaw, J. Z. Gould, P. Tamayo, B. A. Weir, A. Tsherniak, B. Wong, L. A. Garraway, A. F. Shamji, M. A. Palmer, M. A. Foley, W. Winckler, S. L. Schreiber, A. L. Kung, T. R. Golub, Nat. Biotechnol. 2016, 34, 419. 26928769 10.1038/nbt.3460 PMC5508574

[44] Z. E. Perlman, M. D. Slack, Y. Feng, T. J. Mitchison, L. F. Wu, S. J. Altschuler, Science 2004, 306, 1194. 15539606 10.1126/science.1100709

[45] J. Kang, C. H. Hsu, Q. Wu, S. Liu, A. D. Coster, B. A. Posner, S. J. Altschuler, L. F. Wu, Nat. Biotechnol. 2016, 34, 70. 26655497 10.1038/nbt.3419 PMC4844861

[46] B. Raj, D. E. Wagner, A. Mckenna, S. Pandey, A. M. Klein, J. Shendure, J. A. Gagnon, A. F. Schier, Nat. Biotechnol. 2018, 36, 442. 29608178 10.1038/nbt.4103 PMC5938111

[47] C. D. Sago, M. P. Lokugamage, F. Z. Islam, B. R. Krupczak, M. Sato, J. E. Dahlman, J. Am. Chem. Soc. 2018, 140, 17095. 30394729 10.1021/jacs.8b08976 PMC6556374

[48] S. Sun, J. Zhu, X. Zhou, Nat. Methods 2020, 17, 193. 31988518 10.1038/s41592-019-0701-7 PMC7233129

[49] R. Satija, J. A. Farrell, D. Gennert, A. F. Schier, A. Regev, Nat. Biotechnol. 2015, 33, 495. 25867923 10.1038/nbt.3192 PMC4430369

[50] H. L. Sladitschek, U. M. Fiuza, D. Pavlinic, V. Benes, L. Hufnagel, P. A. Neveu, Cell 2020, 181, 922. 32315617 10.1016/j.cell.2020.03.055 PMC7237864

[51] K. H. Chen, A. N. Boettiger, J. R. Moffitt, S. Wang, X. Zhuang, Science 2015, 348, aaa6090. 25858977 10.1126/science.aaa6090 PMC4662681

[52] C. L. Eng, M. Lawson, Q. Zhu, R. Dries, N. Koulena, Y. Takei, J. Yun, C. Cronin, C. Karp, G. C. Yuan, L. Cai, Nature 2019, 568, 235. 30911168 10.1038/s41586-019-1049-y PMC6544023

[53] X. Chen, Y. C. Sun, H. Zhan, J. M. Kebschull, S. Fischer, K. Matho, Z. J. Huang, J. Gillis, A. M. Zador, Cell 2019, 179, 772. 31626774 10.1016/j.cell.2019.09.023 PMC7836778

[54] X. Wang, W. E. Allen, M. A. Wright, E. L. Sylwestrak, N. Samusik, S. Vesuna, K. Evans, C. Liu, C. Ramakrishnan, J. Liu, G. P. Nolan, F. A. Bava, K. Deisseroth, Science 2018, 361, eaat5691. 29930089 10.1126/science.aat5691 PMC6339868

[55] E. Lein, L. E. Borm, S. Linnarsson, Science 2017, 358, 64. 28983044 10.1126/science.aan6827

[56] G. Peng, S. Suo, G. Cui, F. Yu, R. Wang, J. Chen, S. Chen, Z. Liu, G. Chen, Y. Qian, P. Tam, J. J. Han, N. Jing, Nature 2019, 572, 528. 31391582 10.1038/s41586-019-1469-8

[57] L. Li, F. Guo, Y. Gao, Y. Ren, P. Yuan, L. Yan, R. Li, Y. Lian, J. Li, B. Hu, J. Gao, L. Wen, F. Tang, J. Qiao, Nat. Cell Biol. 2018, 20, 847. 29915357 10.1038/s41556-018-0123-2

[58] C. Gu, S. Liu, Q. Wu, L. Zhang, F. Guo, Cell Res. 2019, 29, 110. 30560925 10.1038/s41422-018-0125-4 PMC6355938

[59] J. Goveia, K. Rohlenova, F. Taverna, L. Treps, L. C. Conradi, A. Pircher, V. Geldhof, L. de Rooij, J. Kalucka, L. Sokol, M. García‐Caballero, Y. Zheng, J. Qian, L. A. Teuwen, S. Khan, B. Boeckx, E. Wauters, H. Decaluwé, P. De Leyn, J. Vansteenkiste, B. Weynand, X. Sagaert, E. Verbeken, A. Wolthuis, B. Topal, W. Everaerts, H. Bohnenberger, A. Emmert, D. Panovska, F. De Smet, et al., Cancer Cell 2020, 37, 21. 31935371 10.1016/j.ccell.2019.12.001

[60] B. B. Lake, S. Chen, B. C. Sos, J. Fan, G. E. Kaeser, Y. C. Yung, T. E. Duong, D. Gao, J. Chun, P. V. Kharchenko, K. Zhang, Nat. Biotechnol. 2018, 36, 70. 29227469 10.1038/nbt.4038 PMC5951394

[61] R. Argelaguet, S. J. Clark, H. Mohammed, L. C. Stapel, C. Krueger, C. A. Kapourani, I. Imaz‐Rosshandler, T. Lohoff, Y. Xiang, C. W. Hanna, S. Smallwood, X. Ibarra‐Soria, F. Buettner, G. Sanguinetti, W. Xie, F. Krueger, B. Göttgens, P. J. Rugg‐Gunn, G. Kelsey, W. Dean, J. Nichols, O. Stegle, J. C. Marioni, W. Reik, Nature 2019, 576, 487. 31827285 10.1038/s41586-019-1825-8 PMC6924995

[62] F. Zhou, R. Wang, P. Yuan, Y. Ren, Y. Mao, R. Li, Y. Lian, J. Li, L. Wen, L. Yan, J. Qiao, F. Tang, Nature 2019, 572, 660. 31435013 10.1038/s41586-019-1500-0

[63] N. Rusk, Nat. Methods 2019, 16, 679. 10.1038/s41592-019-0519-3 31363216

[64] M. Stoeckius, C. Hafemeister, W. Stephenson, B. Houck‐Loomis, P. K. Chattopadhyay, H. Swerdlow, R. Satija, P. Smibert, Nat. Methods 2017, 14, 865. 28759029 10.1038/nmeth.4380 PMC5669064

[65] V. M. Peterson, K. X. Zhang, N. Kumar, J. Wong, L. Li, D. C. Wilson, R. Moore, T. K. Mcclanahan, S. Sadekova, J. A. Klappenbach, Nat. Biotechnol. 2017, 35, 936. 28854175 10.1038/nbt.3973

[66] E. P. Mimitou, A. Cheng, A. Montalbano, S. Hao, M. Stoeckius, M. Legut, T. Roush, A. Herrera, E. Papalexi, Z. Ouyang, R. Satija, N. E. Sanjana, S. B. Koralov, P. Smibert, Nat. Methods 2019, 16, 409. 31011186 10.1038/s41592-019-0392-0 PMC6557128

[67] A. Crinier, P. Milpied, B. Escalière, C. Piperoglou, J. Galluso, A. Balsamo, L. Spinelli, I. Cervera‐Marzal, M. Ebbo, M. Girard‐Madoux, S. Jaeger, E. Bollon, S. Hamed, J. Hardwigsen, S. Ugolini, F. Vély, E. Narni‐Mancinelli, E. Vivier, Immunity 2018, 49, 971. 30413361 10.1016/j.immuni.2018.09.009 PMC6269138

[68] X. Han, R. Wang, Y. Zhou, L. Fei, H. Sun, S. Lai, A. Saadatpour, Z. Zhou, H. Chen, F. Ye, D. Huang, Y. Xu, W. Huang, M. Jiang, X. Jiang, J. Mao, Y. Chen, C. Lu, J. Xie, Q. Fang, Y. Wang, R. Yue, T. Li, H. Huang, S. H. Orkin, G. C. Yuan, M. Chen, G. Guo, Cell 2018, 172, 1091. 29474909 10.1016/j.cell.2018.02.001

[69] J. D. Buenrostro, M. R. Corces, C. A. Lareau, B. Wu, A. N. Schep, M. J. Aryee, R. Majeti, H. Y. Chang, W. J. Greenleaf, Cell 2018, 173, 1535. 29706549 10.1016/j.cell.2018.03.074 PMC5989727

[70] A. Regev, S. A. Teichmann, E. S. Lander, I. Amit, C. Benoist, E. Birney, B. Bodenmiller, P. Campbell, P. Carninci, M. Clatworthy, H. Clevers, B. Deplancke, I. Dunham, J. Eberwine, R. Eils, W. Enard, A. Farmer, L. Fugger, B. Göttgens, N. Hacohen, M. Haniffa, M. Hemberg, S. Kim, P. Klenerman, A. Kriegstein, E. Lein, S. Linnarsson, E. Lundberg, J. Lundeberg, P. Majumder, et al., Elife 2017, 6, e27041. 29206104 10.7554/eLife.27041 PMC5762154

[71] X. Han, Z. Zhou, L. Fei, H. Sun, R. Wang, Y. Chen, H. Chen, J. Wang, H. Tang, W. Ge, Y. Zhou, F. Ye, M. Jiang, J. Wu, Y. Xiao, X. Jia, T. Zhang, X. Ma, Q. Zhang, X. Bai, S. Lai, C. Yu, L. Zhu, R. Lin, Y. Gao, M. Wang, Y. Wu, J. Zhang, R. Zhan, S. Zhu, et al., Nature 2020, 581, 303. 32214235 10.1038/s41586-020-2157-4

[72] N. Aizarani, A. Saviano, Sagar, L. Mailly, S. Durand, J. S. Herman, P. Pessaux, T. F. Baumert, D. Grün, Nature 2019, 572, 199. 31292543 10.1038/s41586-019-1373-2 PMC6687507

[73] J. S. Herman, Sagar, D. Grün, Nat. Methods 2018, 15, 379. 29630061 10.1038/nmeth.4662

[74] M. Asp, S. Giacomello, L. Larsson, C. Wu, D. Fürth, X. Qian, E. Wärdell, J. Custodio, J. Reimegård, F. Salmén, C. österholm, P. L. Ståhl, E. Sundström, E. åkesson, O. Bergmann, M. Bienko, A. Månsson‐Broberg, M. Nilsson, C. Sylvén, J. Lundeberg, Cell 2019, 179, 1647. 31835037 10.1016/j.cell.2019.11.025

[75] S. Giacomello, F. Salmén, B. K. Terebieniec, S. Vickovic, J. F. Navarro, A. Alexeyenko, J. Reimegård, L. S. Mckee, C. Mannapperuma, V. Bulone, P. L. Ståhl, J. F. Sundström, N. R. Street, J. Lundeberg, Nat. Plants 2017, 3, 17061. 28481330 10.1038/nplants.2017.61

[76] P. L. Ståhl, F. Salmén, S. Vickovic, A. Lundmark, J. F. Navarro, J. Magnusson, S. Giacomello, M. Asp, J. O. Westholm, M. Huss, A. Mollbrink, S. Linnarsson, S. Codeluppi, Å. Borg, F. Pontén, P. I. Costea, P. Sahlén, J. Mulder, O. Bergmann, J. Lundeberg, J. Frisén, Science 2016, 353, 78. 27365449 10.1126/science.aaf2403

[77] R. Ke, M. Mignardi, A. Pacureanu, J. Svedlund, J. Botling, C. Wählby, M. Nilsson, Nat. Methods 2013, 10, 857. 23852452 10.1038/nmeth.2563

[78] W. Chung, H. H. Eum, H. O. Lee, K. M. Lee, H. B. Lee, K. T. Kim, H. S. Ryu, S. Kim, J. E. Lee, Y. H. Park, Z. Kan, W. Han, W. Y. Park, Nat. Commun. 2017, 8, 15081. 28474673 10.1038/ncomms15081 PMC5424158

[79] S. V. Puram, I. Tirosh, A. S. Parikh, A. P. Patel, K. Yizhak, S. Gillespie, C. Rodman, C. L. Luo, E. A. Mroz, K. S. Emerick, D. G. Deschler, M. A. Varvares, R. Mylvaganam, O. Rozenblatt‐Rosen, J. W. Rocco, W. C. Faquin, D. T. Lin, A. Regev, B. E. Bernstein, Cell 2017, 171, 1611. 29198524 10.1016/j.cell.2017.10.044 PMC5878932

[80] S. Cheng, Z. Li, R. Gao, B. Xing, Y. Gao, Y. Yang, S. Qin, L. Zhang, H. Ouyang, Du P, L. Jiang, B. Zhang, Y. Yang, X. Wang, X. Ren, J. X. Bei, X. Hu, Z. Bu, J. Ji, Z. Zhang, Cell 2021, 184, 792. 33545035 10.1016/j.cell.2021.01.010

[81] J. S. Lee, S. Park, H. W. Jeong, J. Y. Ahn, S. J. Choi, H. Lee, B. Choi, S. K. Nam, M. Sa, J. S. Kwon, S. J. Jeong, H. K. Lee, S. H. Park, S. H. Park, J. Y. Choi, S. H. Kim, I. Jung, E. C. Shin, Sci. Immunol. 2020, 5, eabd1554. 32651212 10.1126/sciimmunol.abd1554 PMC7402635

[82] F. Zhang, R. Gan, Z. Zhen, X. Hu, X. Li, F. Zhou, Y. Liu, C. Chen, S. Xie, B. Zhang, X. Wu, Z. Huang, Signal Transduction Targeted Ther. 2020, 5, 156. 10.1038/s41392-020-00263-y PMC7426596 32796814

[83] J. Y. Zhang, X. M. Wang, X. Xing, Z. Xu, C. Zhang, J. W. Song, X. Fan, P. Xia, J. L. Fu, S. Y. Wang, R. N. Xu, X. P. Dai, L. Shi, L. Huang, T. J. Jiang, M. Shi, Y. Zhang, A. Zumla, M. Maeurer, F. Bai, F. S. Wang, Nat. Immunol. 2020, 21, 1107. 32788748 10.1038/s41590-020-0762-x

